# Anæsthetics

**Published:** 1892-06-11

**Authors:** 


### The Doctor's Armchair.

### ANÆSTHETICS.

During the five years beginning with 1887 and ending with 1891, there were seventy-two deaths from anæsthetics in this country. Of these, three were caused by what is called the A.C.E. mixture, a mixture of alcohol one part, chloroform two parts, and ether three parts. Four resulted from ether alone, and the remaining sixty-five were due to chloroform. We take these figures from a little book, written by Mr. Henry Davis, teacher and administrator of anæsthetics at St. Mary's Hospital. The full title of the book is "A Guide to the Administration of Anæsthetics," and its publisher is Mr. H. K. Lewis, of Gower Street.

If the reader will bear in mind the figure seventy-two as the number of deaths that have occurred from anæsthetics during the last five years, and will at the same time weigh carefully the following sentences from Mr. Davis' book, he will not, if he be a doctor of brains and conscience, feel very comfortable in his mind. "These facts," says Mr. Davis, "beyond showing that the inhalation of chloroform is attended with considerable risk, do not help us much, as we have no evidence to guide us as to the actual number of times chloroform was administered. An examination of the cases operated upon shows that many were of a simple nature—*e.g.*, opening of abscesses, phymosis, removal of fingers, extraction of teeth, and the like—operations which could have been equally well performed with ether or even gas. Indeed, *in more than half the fatal cases from chloroform*, there appears to have been no good reason to employ chloroform at all in preference to ether. Of the sixty-five fatal cases from chloroform, no fewer than six occurred in patients suffering from pleural effusion or empyema; two of the fatal cases occurred during delivery, and three when unconsciousness was induced for the reduction of dislocated humeri."

Now the full meaning of this passage is that more than half the sixty-five cases of death from chloroform were the result either of ignorance or of wilfulness on the part of those who selected the most powerful and the most dangerous anæsthetic for cases where a less powerful and a less dangerous substitute would have been sufficient for all practical purposes. This is a condition of things which, whatever may be its effect upon members of the medical profession when they hear of it, will give the public a most unpleasant shock. The non-medical world has been industriously and perseveringly taught for many years to believe that in hospitals, if anywhere, human life is regarded with the maximum of sacredness. There, if anywhere, the highest scientific intelligence, combined with the most trained and finished operative skill, is brought to bear upon the case of every man, woman, and child. Such a thing as insufficient knowledge, careless inexperience, and vulgar indifference to life is believed by many to be as foreign to hospitals as lies on a death-bed or fraud in heaven. Mr. Davis appears to know better, and many of us who are not anæsthetic specialists, but who yet have had much experience of actual hospital life, know better, too. There are a good many house surgeons, and not a few nurses, who, if their consciences were not asleep, would read some parts of Mr. Davis' little book with something like terror. Such are the things that have been seen in the past; such must not be the things that are to be seen in the future.

Many house surgeons, and many senior students and nurses, no doubt consider the administration of an anæsthetic as the least important part of the work of an operation day. They will not think so if they read Mr. Davis' book. The author magnifies his office; and it is most fitting that he should do so. If he magnifies it unduly, the undueness consists in magnifying it too little rather than too much. What experienced medical man would willingly place himself so close to the very edge of the precipice of death as every patient is placed who is put thoroughly under the influence of a powerful anæsthetic? There is hardly one man in a hundred who, if he were as fully conscious as he is profoundly unconscious, of his nearness to death when deeply anæsthetised, would not slip finally over the precipice from sheer fright. And yet many operating surgeons never give so much as a thought as to whether the cases they are about to operate upon imperatively demand the profound anæsthesia of chloroform, or can be safely manipulated under the much less potent influence of ether, or even under the harmless gas.

The art of medicine, we are always insisting, should be thoroughly taught in hospitals; the art as distinguished from the science, though not without the science—the art in its fundamentally important details. No experienced medical man will deny that hospital students, even at the present time, are left to pick up much of the art of daily medicine and the details of practice in any way they can. Take, for example, this very case of the administration of anæsthetics! Whoever heard of a clinical lecture upon the subject? What clinical tutor ever made it a part of his systematic teaching? How many text-books had been written on anæsthetics ten years ago? Are there so many as half-a-dozen even to-day?

It is impossible to help feeling that, with all the thoroughness of our modern hospitals, there is still the possibility of a great deal of slip-shod work being done by house surgeons, students, nurses, and even by older officials. But this slip-shod work has to do with human lives, and in the department of the administration of anæsthetics alone it seems to result in at least thirty unnecessary deaths every five years—far more, no doubt, if all the facts were known—but thirty at least. To examine your operation patient thoroughly beforehand; to duly prepare him as to his food and as to his clothing; to consider the nature and probable duration of the operation; and to make selection of such an anæsthetic as will answer all practical purposes, with the minimum of danger to the patient's life—these are, surely, the very rudiments and A B C of surgery and anæsthetics. These are the things that are taught, and well taught, in Mr. Davis' little book; and these are the things which every house surgeon, student, and nurse should learn, there or elsewhere.

